# New aryloxybenzylidene ruthenium chelates – synthesis, reactivity and catalytic performance in ROMP

**DOI:** 10.3762/bjoc.11.206

**Published:** 2015-10-14

**Authors:** Patrycja Żak, Szymon Rogalski, Mariusz Majchrzak, Maciej Kubicki, Cezary Pietraszuk

**Affiliations:** 1 Adam Mickiewicz University in Poznań, Faculty of Chemistry, Umultowska 89b, 61-614 Poznań, Poland

**Keywords:** chemoactivation, latent catalysts, metathesis, ROMP, ruthenium

## Abstract

New phenoxybenzylidene ruthenium chelates were synthesised from the second generation Grubbs catalysts bearing a triphenylphosphine ligand (or its para-substituted analogues) by metathesis exchange with substituted 2-vinylphenols. The complexes behave like a latent catalyst and are characterized by an improved catalytic behaviour as compared to that of the known analogues, i.e., they exhibit high catalytic inactivity in their dormant forms and a profound increase in activity after activation with HCl. The strong electronic influence of substituents in the chelating ligand on the catalytic activity was demonstrated. The catalytic properties were tested in ROMP of cyclooctadien (COD) and a single selected norbornene derivative.

## Introduction

Olefin metathesis is nowadays one of the most important methods for the formation of carbon–carbon bonds in organic and polymer chemistry [1–2]. The availability of well-defined ruthenium-based catalysts, showing a number of desirable features such as tolerance of functional groups, moisture and air, has significantly expanded the scope and application of this process regardless of dynamic advancement in the development of ruthenium-based metathesis catalysts. Continuous efforts have been aimed at the search for new catalysts characterized by improved stability and catalytic performance. One of the current challenges is the development of catalysts allowing control of initiation for some metathesis polymerisation processes. For such applications a variety of latent catalysts have been reported which permit control of initiation and efficient propagation of the reaction [3–5].

**Figure 1:**
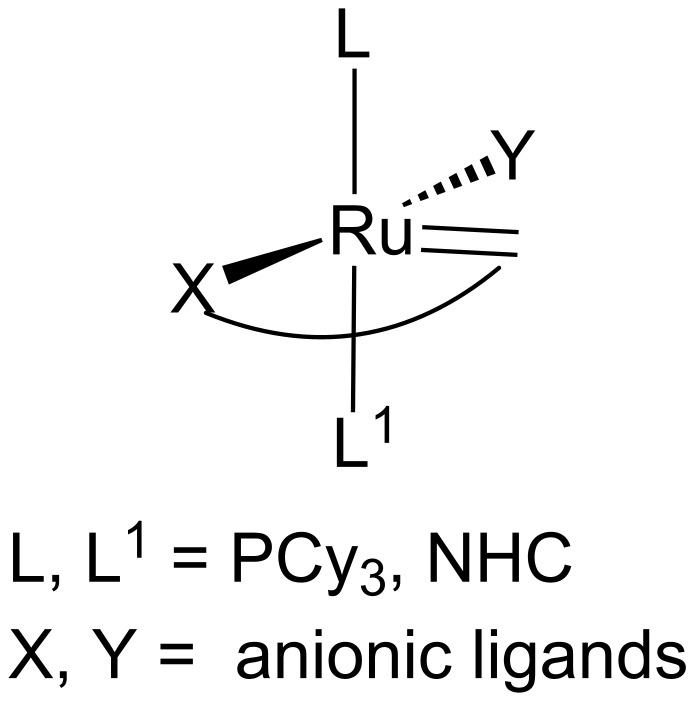
Coordination motif of latent catalyst of olefin metathesis in which alkylidene ligand is bound to the heteroatom X, acting as an anionic ligand.

**Figure 2:**
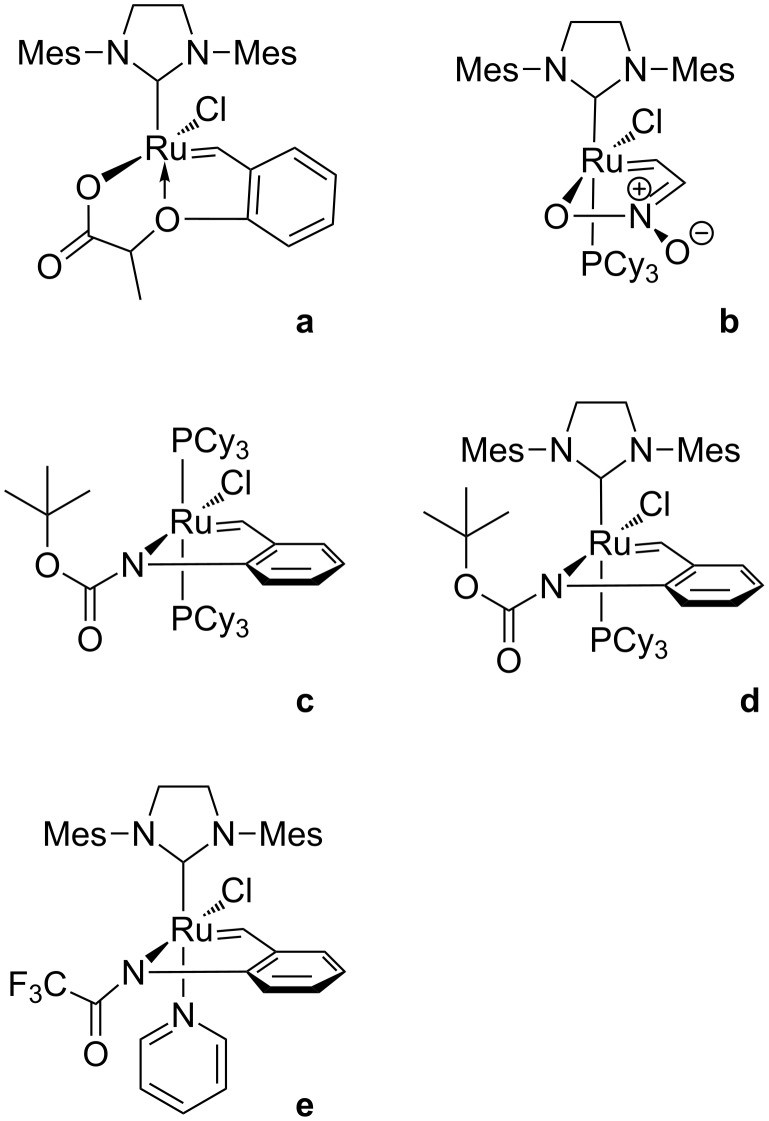
Known latent catalysts of olefin metathesis in which alkylidene ligands are bound to a heteroatom, acting as an anionic ligand.

Among numerous examples of latent catalysts, the complexes representing the structural motif illustrated in Figure 1 have been relative poorly investigated. Known examples include benzylidenecarboxylate (Figure 2a) [6] and nitronate complexes (Figure 2b) [7] as well as amidobenzylidene ruthenium chelates (Figure 2c–e) that we have disclosed in cooperation with the Grela group [8].

**Figure 3:**
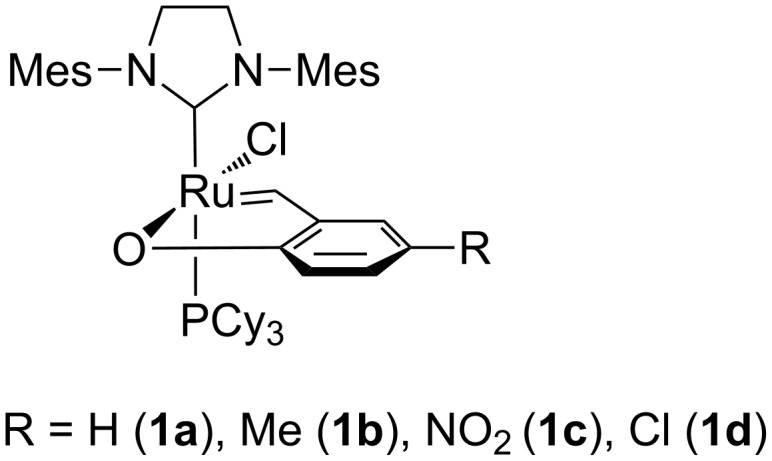
Selected, known aryloxybenzylidene chelates [9–10].

Recently we have reported a study on aryloxybenzylidene ruthenium chelates (**1a–d** Figure 3) [9]. Similar complexes have also been independently studied by Skowerski and Grela [10].

Phenoxybenzylidene complexes have been demonstrated to behave like latent catalysts in common testing reactions involving ring opening metathesis polymerisation (ROMP) of COD, norbornene derivative and dicyclopentadiene as well as cross metathesis (CM) of allylbenzene with *Z*-1,4-(diacetoxy)but-2-ene [9–10]. Although catalysts **1a**–**d** are characterized by a number of advantages, they are not free from some weaknesses. They showed in some reactions a non-negligible catalytic activity in the absence of activators [9–10] and an instability of the activated forms. Herein, searching for improved latent catalysts that are less reactive in dormant form and highly active in the presence of an chemical activator, we report the synthesis and catalytic performance of new phenoxybenzylidene ruthenium chelates modified by introduction of electron donating and electron withdrawing substituents at the benzylidene ligand in para position to the coordinating oxygen and bearing instead of a strong sigma-donor ligand – tricyclohexylphosphine a weaker sigma-donor – a triphenylphosphine ligand or its derivatives. The catalytic performance of the synthesized complexes were tested in ROMP of COD and a single selected norbornene derivative.

## Results and Discussion

### Synthesis

**Scheme 1:**
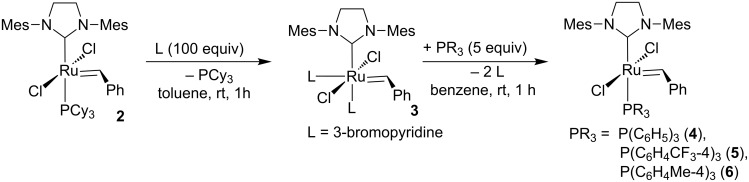
Synthesis of catalyst precursors **4–6** [11].

Complexes containing triphenylphosphine ligands and substituted triphenylphosphine ligands were isolated in high yields (95–98%) according to the methodology described by Grubbs (Scheme 1) [11]. However, in our hands to get complete transformation 5 equiv of phosphine had to be used.

**Scheme 2:**
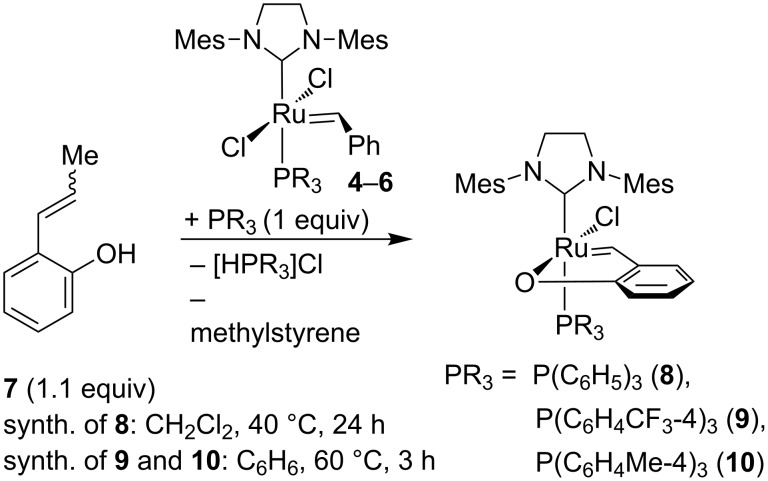
Synthesis of catalysts **8–10**.

In a next step the complexes (**4**–**6**) were subjected to metathesis exchange reaction with 2-(prop-1-enyl)phenol (Scheme 2). The reaction was performed in the presence of an equimolar amount of the corresponding phosphine in order to bind the HCl liberated during the reaction, which resulted in a significant increase in the reaction yield. Complexes **8**–**10** were easily isolated by precipitation with methanol or hexane from concentrated solution in methylene chloride (isolated yields = 86–90%). ^1^H NMR spectra confirmed the formation of new alkylidene complexes.

**Scheme 3:**
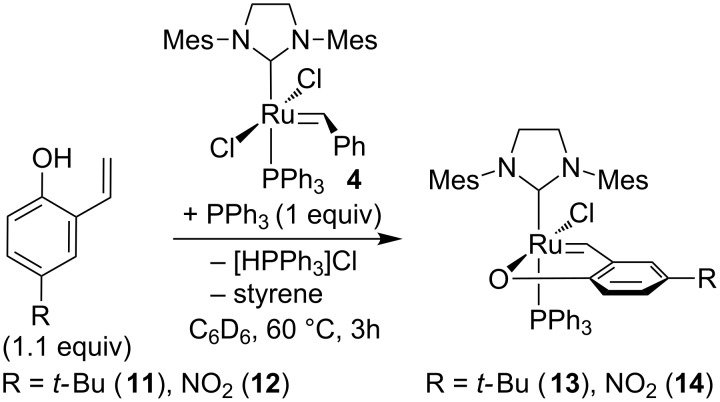
Synthesis of catalysts **13** and **14**.

Complexes **13** and **14** were prepared by using a similar methodology. Complex **4** was subjected to a metathesis exchange with a slight excess of the appropriate 2-vinylphenol in the presence of triphenylphosphine (Scheme 3). Complexes were obtained with isolated yields of 90% (complex **13**) and 92% (complex **14**).

### Catalytic activity

**Figure 4:**
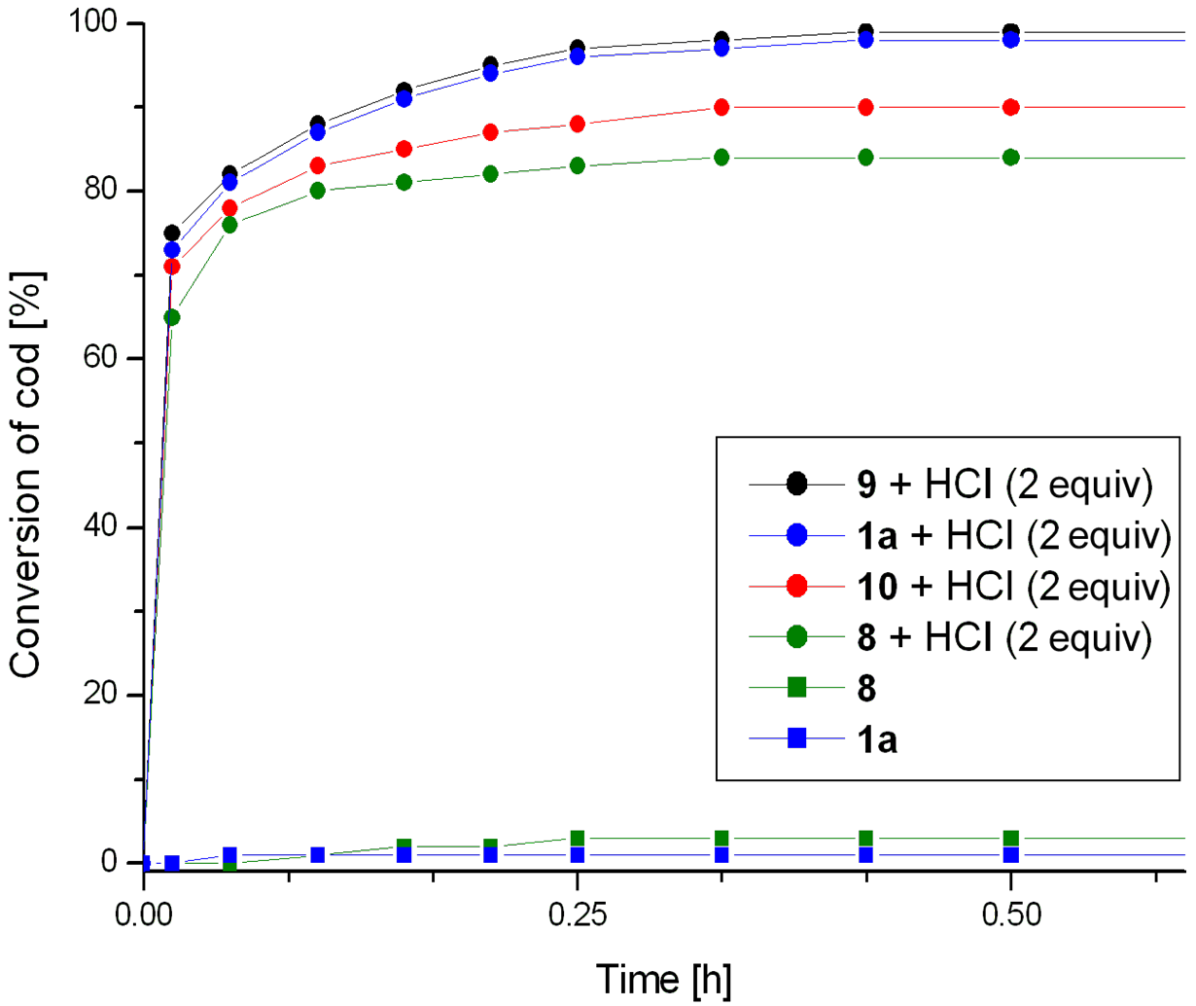
ROMP of COD. Conditions: CH_2_Cl_2_, 40 °C, 0.5 M, [COD]:[Ru] = 20000; For clarity, only two representative profiles for non-activated catalysts were presented.

The obtained complexes were tested in the ring opening metathesis polymerisation (ROMP) of cyclooctadiene. First, the impact of a phosphine ligand on the catalytic activity was investigated. Preliminary tests performed showed, as expected, that in the absence of the activator complexes **1a** and **8**–**10** were completely inactive (CH_2_Cl_2_, 40 °C, 0.5 M, 0.1 mol % of catalyst). Under the same conditions, in the presence of 2 equivalents of HCl (used in the form of 2.0 M solution in diethyl ether) as an activating agent, complex **1a** was capable for providing a complete conversion after a few minutes of the reaction. Preliminary tests to optimize the concentration of the catalyst showed that complex **1a** in the presence of an activator retained high catalytic activity in the test reaction already at a concentration of 0.005 mol % (relative to the monomer). The reaction profiles for the catalysts **1a** and **8**–**10**, both in the dormant form and in the presence of an activator are shown in Figure 4. The results confirm the total lack of activity of complexes in their dormant forms and show a dramatic increase in catalytic activity in the presence of 2 equiv of HCl as an activator. The chart illustrates an insignificant effect of the properties of the phosphine ligand on the catalytic activity of the complexes. The order of increasing activities of the activated species, i.e., **8** (PPh_3_) < **10** P(C_6_H_4_Me-4)_3_ < **1a** (PCy_3_) < **9** P(C_6_H_4_CF_3_-4)_3_ does not correlate with decreasing σ-donor ability of the phosphine ligands (represented by Hammet constant) [12–13]. The highest activity was indeed observed for complex **9** containing the weakest σ-donor and potentially most easily dissociating ligand P(C_6_H_4_CF_3_-4)_3_. However, the lowest activity was found for complex **8** containing triphenylphosphine which is characterised by lower σ-strength than a tris(*p*-tolyl)phosphine present in catalyst **10** and tricyclohexylphosphine present in the very active catalyst **1a**. The increase in activity could be correlated to some extent with growing basicity of the phosphine ligand. More basic phosphine more readily reacted with the activator (HCl) leading to a faster increase in the concentration of the phosphine-free form of the catalyst (Figure 4).

**Figure 5:**
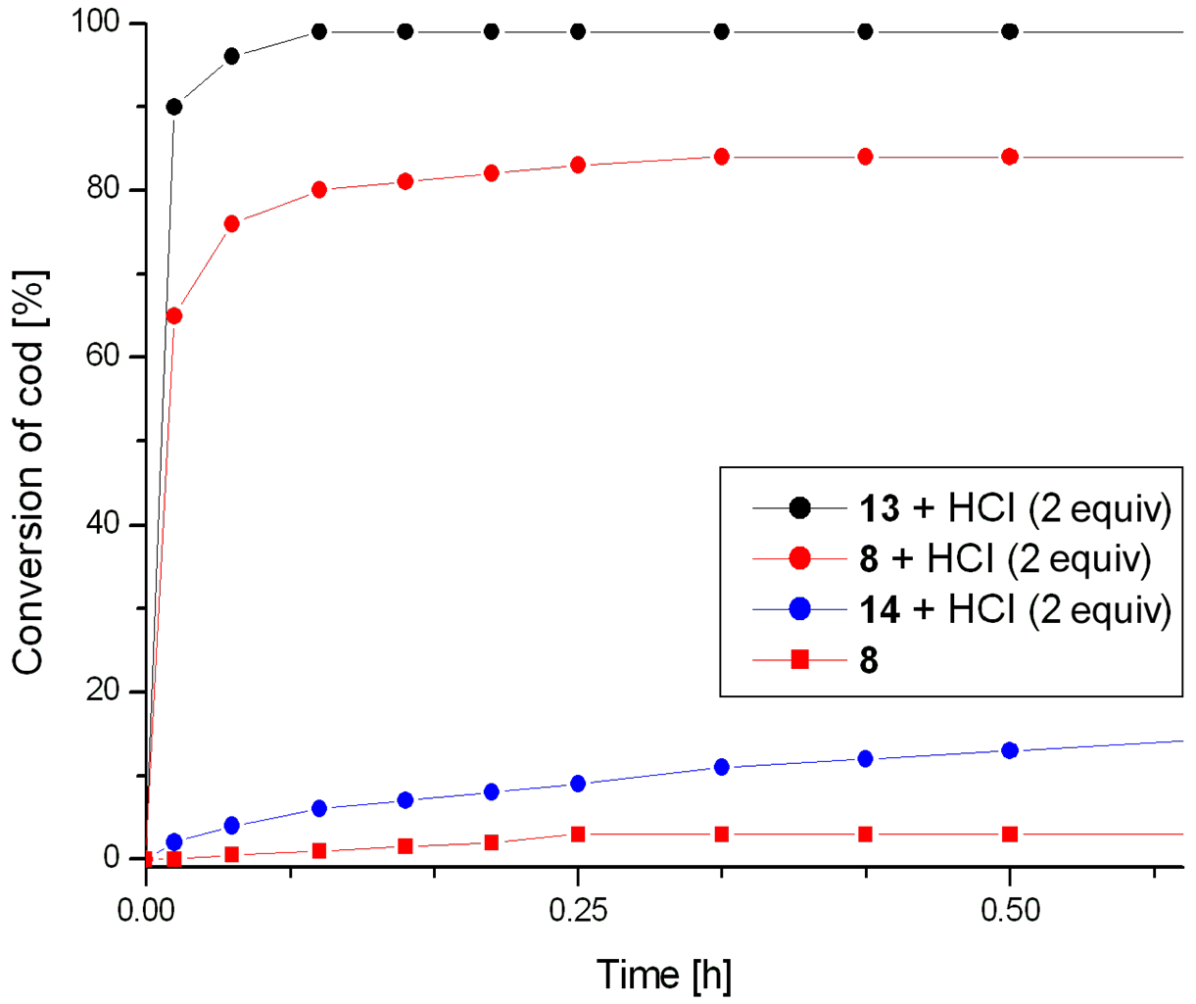
ROMP of cod. Conditions: CH_2_Cl_2_, 40 °C, 0.5 M, [cod]:[Ru] = 20000; For clarity only representative profile for non-activated catalyst is presented.

The influence of electronic properties of substituents placed in the aromatic ring of the chelating ligand, in para position to the oxygen covalently bound to the ruthenium atom, was examined by comparing the activity of complexes **8**, **13** and **14**. Complexes **13** and **14**, when used without any activating additives, showed no catalytic activity under the reaction conditions. However, in the presence of two equivalents of HCl, the effect of the electronic properties of the above substituents was significant. Comparison of the activities of catalysts **8**, **13** and **14** in their dormant forms and in the presence of an activator is shown in Figure 5. The highest activity was observed for catalyst **13** containing an electron donating *tert*-butyl group at the aromatic ring. In the presence of this catalyst, the addition of the activator resulted in a dramatic increase in catalytic activity, so that complete conversion was observed after a few minutes of the reaction course. On the other hand, the lowest activity was observed for complex **14** containing a strongly electron withdrawing nitro group. In the presence of this catalyst, after 1 h of the reaction only about 17% conversion took place. A similar impact of substituents was observed for complexes **1a**–**c** [9]. A reasonable explanation of the activating influence of electron donating groups is an increase in electron density on the chelating oxygen atom generated by a positive inductive effect, which facilitates the protonolysis of the Ru–O bond. The strongly electron withdrawing nitro group present in complex **14** caused reduction of the electron density on the oxygen atom and consequently its lower susceptibility to protonolysis which has been earlier proved to be necessary for the catalyst activation [9].

**Scheme 4:**
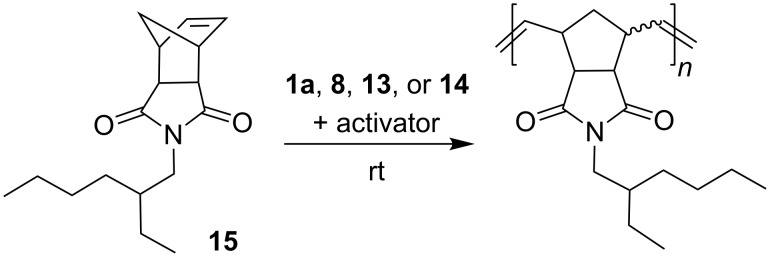
ROMP of monomer **15**.

The catalytic performance of phenoxybenzylidene ruthenium chelates **1a**, **8**, **13** and **14** was also checked in ROMP of a single selected norbornene derivative **15** (Scheme 4). The reaction progress was monitored by ^1^H NMR spectroscopy.

**Figure 6:**
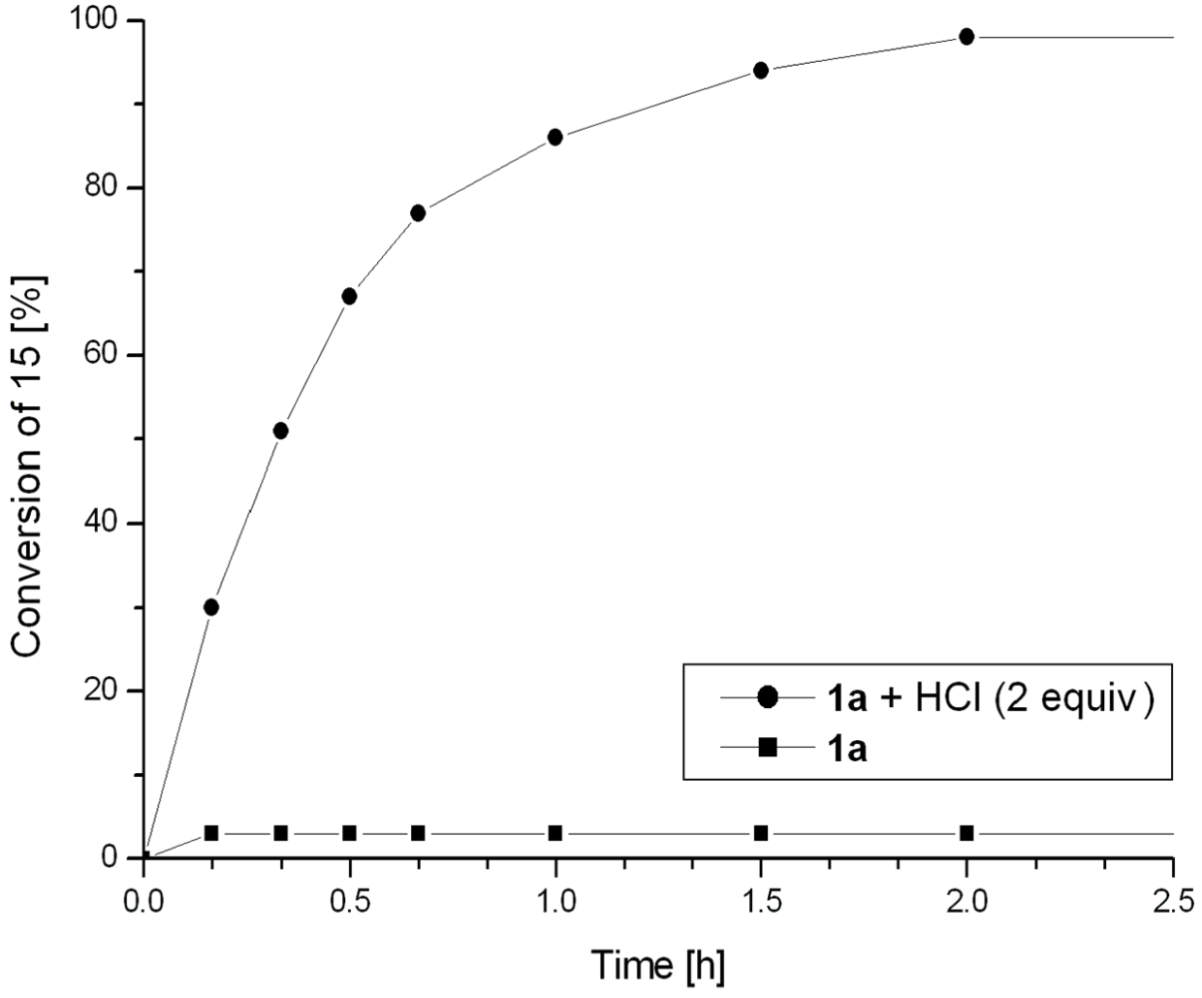
ROMP of monomer **15**. Conditions: CDCl_3_, 40 °C, 0.08 M, [**15**]:[Ru] = 200.

Inactivated catalyst **1a** does not exhibit catalytic activity in ROMP of **15** performed at room temperature (23 °C). When HCl is added, the activity of complex **1a** increases, but after 2 h of the reaction course only about 30% monomer conversion was observed. At 40 °C, complex **1a** used without an activator remained inactive, but the addition of HCl led to complete monomer conversion within 2 h (Figure 6). In a separate experiment performed at room temperature the activated complex **1a** gave 90% monomer conversion within 2 h, by using a monomer to catalyst ratio as high as 2000.

**Figure 7:**
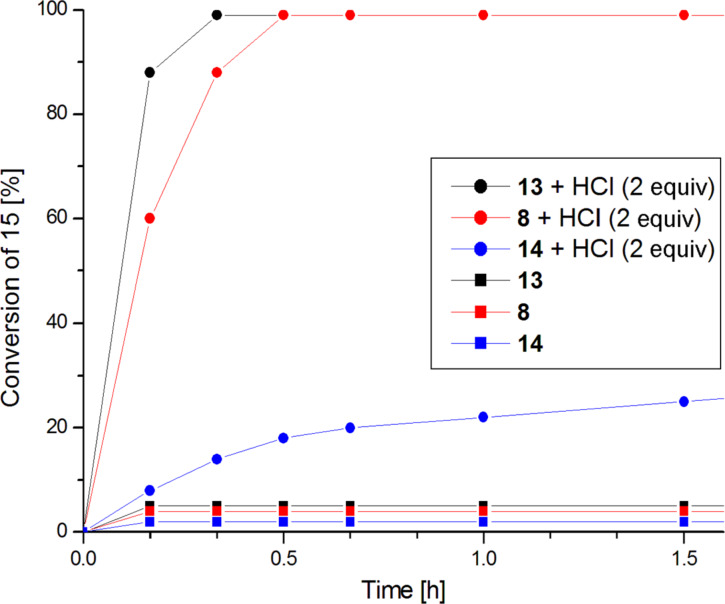
ROMP of monomer **15**. Conditions: CDCl_3_, 23 °C, 0.08 M, [**15**]:[Ru] = 200.

Preliminary studies of ROMP of monomer **15** in the presence of activated complexes **8**, **13** and **14** revealed their significantly higher catalytic activity than that of activated complex **1a**; that is why a further study of ROMP was performed at room temperature. In the absence of an activator, the reaction over all these complexes gave only trace monomer conversion (Figure 7). After the activation with 2 equiv HCl, complete monomer conversion was observed to occur within 30 min in the presence of catalyst **8** and within 20 min for catalyst **13**. Complex **14** exhibits significantly lower activity. After 1 h only 22% conversion was noted and complete monomer consumption required 24 h of the reaction course.

### Activation process study

**Scheme 5:**
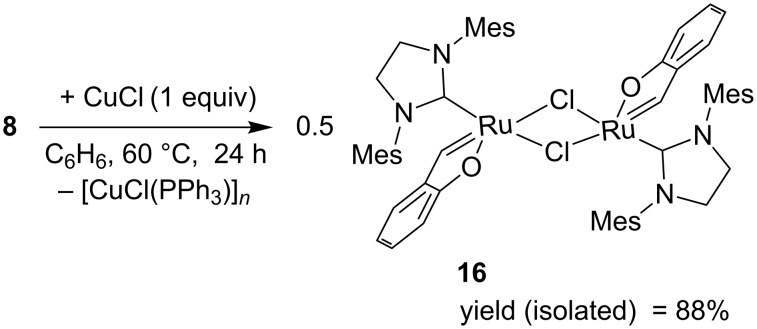
Formation of phosphine free dimeric complex in the presence of CuCl.

**Figure 8:**
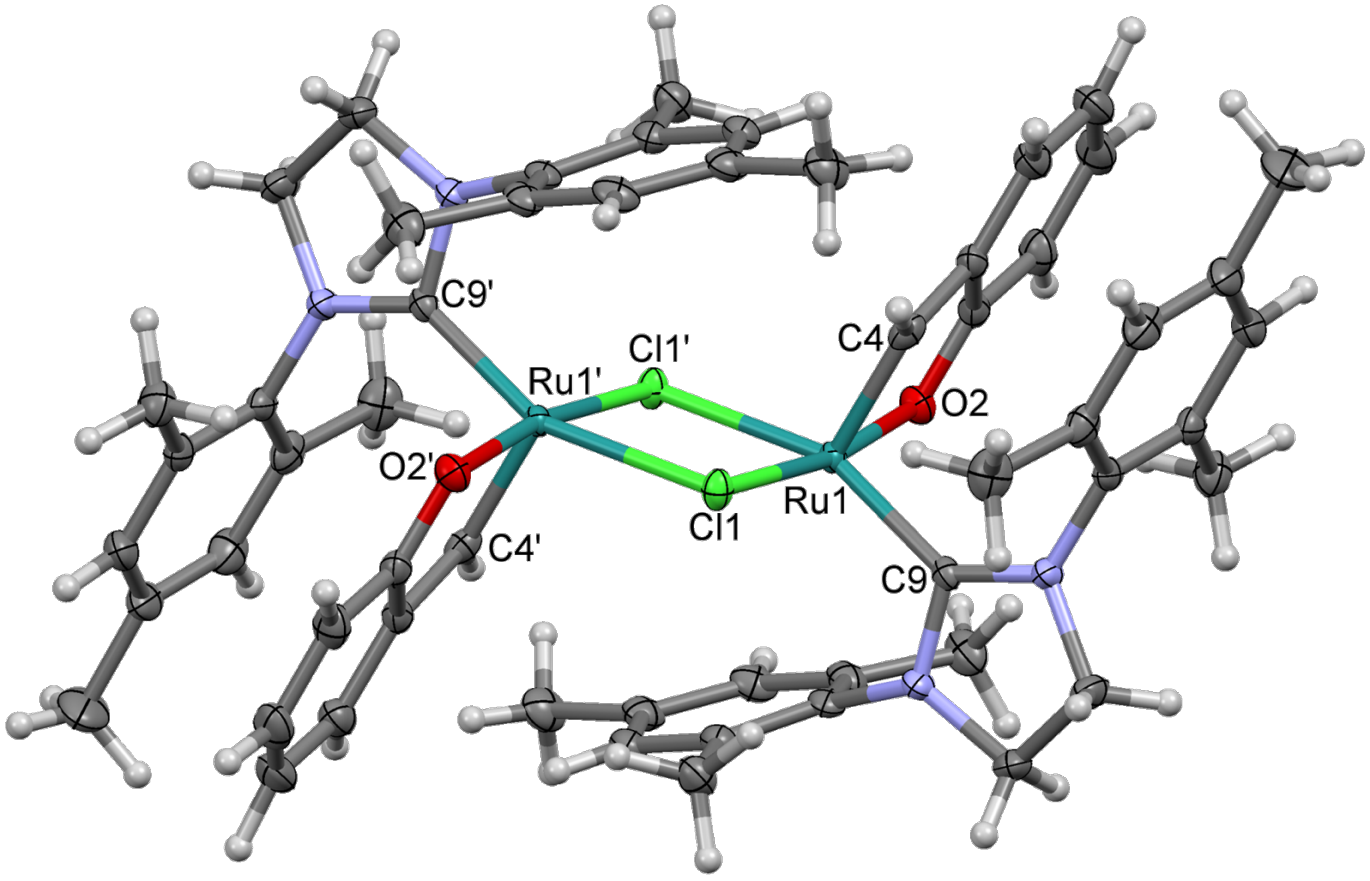
A perspective view of complex **16**, the ellipsoids are drawn at the 50% probability level. Hydrogen atoms are drawn as spheres of arbitrary radii.

Our earlier studies of the activation of complex **1a** with an ethereal solution of HCl have proved that the chelate ring opening by cleavage of the Ru–O bond is necessary for getting the catalytically active form of this complex [9]. On the other hand, the studies of the effect of CuCl, acting as a phosphine scavenger, on the activity of complex **1a** in ROMP of COD revealed a small activating effect [9]. An analogous study performed for ROMP of COD catalysed with complex **8** did not confirm the activating impact of CuCl. Complex **8** used alone or in the presence of 2–5 equiv of CuCl was totally inactive. In order to elucidate the possible transformations taking place in the system phenoxybenzylidene chelate/CuCl, a benzene solution of complex **8** was heated with 1 equiv of CuCl at 60 °C. After 24 h of the reaction course, the formation of a green precipitate was observed. X-ray diffraction analysis of single crystals obtained by slow evaporation of the post-reaction mixture revealed the formation of dimeric complex **16**, in which the phenoxybenzylidene ring was conserved (Figure 8), which points to the reaction proceeding according to Scheme 5.

Similar transformation was observed with the use of complex **1a** as a starting compound [9]. The reaction was particularly effective in benzene because of poor solubility of the dimer in this solvent.

**Scheme 6:**
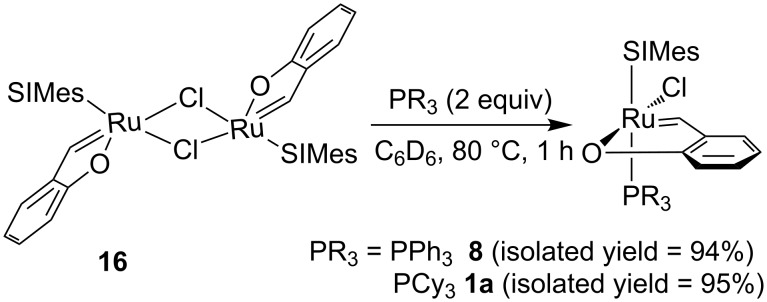
Synthesis of complexes **1a** and **8** starting from dimer **16**.

As it is common for ruthenium–alkylidene complexes active in metathesis, the coordination of the Ru atom might be described as a distorted tetragonal pyramid, with the carbon atom double-bonded to Ru (C4) in the apex of the pyramid. The distortions in the dimer are more pronounced than in the similar mononuclear complex **1a** (see supplemetara data of [9]), but still four base atoms; two chlorines, carbon C9 and oxygen O2 are planar within ca. 0.22 Å, Ru lies also quite well within this plane (0.17Å), and only the C4 atom is by 1.99 Å distant from the plane. The Ru–C line makes an angle of ca. 8.6° with the normal to the mean basal plane. The double-bond Ru1–C4 (1.841(3) Å) is significantly shorter than the formally single Ru–C9 bond of 1.974(2) Å. Probably due to the steric requirements, Ru–Cl distances in the dimer are longer than in mononuclear complex **1a** (2.4145(5) and 2.42775(5) Å, as compared to 2.3827(4)), while the other bonds are slightly (Ru=C: 1.841(3) vs 1.8499(18) Å, Ru–O: 2.0720(16) vs 2.0936(12) or significantly (Ru–O: 1.974(2) vs 2.0720(16)) shorter. Complex **16** showed no catalytic activity in the ROMP of COD under the conditions used (CH_2_Cl_2_, 40 °C, 0.5 M, 0.005–0.1 mol % relative to the monomer). The observed catalytic inactivity was found not to be a consequence of conservation of chelating phenoxybenzylidene ring in the dimer structure but results from the fast decomposition of complex **16** in solution, leading to the loss of alkylidene moiety. However, when a suspension of dimer **16** in CH_2_Cl_2_ was treated with an equimolar amount of triphenylphosphine formation of complex **8** with an almost quantitative yield was observed. A similar reaction was observed when strongly nucleophilic PCy_3_ was used (Scheme 6). The reaction can be successfully used as an alternative method for the synthesis of a variety of phenoxybenzylidene chelates (see Experimental).

**Scheme 7:**
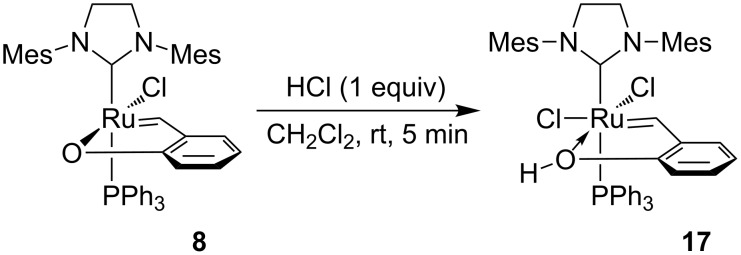
Activation of complex **8** with one equivalent of HCl.

According to the earlier described activation mechanism [9], the reaction of phenoxybenzylidene chelates with 1 equiv of HCl leads to protonation of the oxygen atom in the Ru–O bond, which results in the breaking of the bond and introduction of a chloride anion into the coordination sphere of ruthenium. Exposition of complex **8** to one equivalent of HCl brought a change in the solution colour from green to light-green. The fine-crystalline light-green precipitate was isolated from the post-reaction mixture by precipitation with hexane with 95% isolated yield. The precipitate was stable as solid and sufficiently stable in a CD_2_Cl_2_ solution to permit recording of its ^1^H and ^31^P NMR spectra. On the basis of the data obtained the reaction was proposed to proceed according to Scheme 7. However, it was impossible to identify in the ^1^H NMR spectrum the signal that could be assigned to the hydroxy group. It is most probably a consequence of high lability of this proton and its suitability for exchange with deuterium coming from the NMR solvent (CD_2_Cl_2_) as it was observed for activated complex **1a** [9].

Complex **17** was proved to exhibit high catalytic activity in ROMP of the monomers tested. In ROMP of COD (CH_2_Cl_2_, 40 °C, 0.5 M, [COD]: [Ru] = 20000) it permits obtaining complete conversion after 10 min of the reaction course. Performed tests of ROMP of monomer **15** showed complete conversion within 15 min (CDCl_3_, 23 °C, 0.08 M, [**15**]:[Ru] = 200) and within 1 h when using monomer to catalyst ratio equal to 2000.

## Conclusion

Ruthenium–benzylidene complexes bearing a triphenylphosphine ligand or its para-substituted analogues undergo metathetic exchange with 2-(prop-1-enyl)phenol or substituted 2-vinylphenols to form phenoxybenzylidene ruthenium chelates. These complexes in the phenoxide form exhibit nearly no activity in ROMP of COD and an exemplary norbornene derivative. However, they can be easily activated by addition of an ethereal solution of HCl. The catalytic activity in their active forms was found to be related to the basicity and nucleophilicity of the phosphine ligands. A strong electronic influence of the substituent in the ring of the phenoxybenzylidene ligand, in para position towards the oxygen atom, on the catalytic activity of the active form of the complexes was found. The presence of an electron-donating *tert*-butyl substituent gave a significant increase in the complex activity, while in the presence of a strongly electron-accepting nitro group the strong opposite effect was observed. When compared to the earlier described analogous complexes, the new phenoxybenzylidene chelates exhibit profound catalytic inactivity in their dormant forms and an improved catalytic activity (after activation) in ROMP of tested monomers.

## Experimental

See Supporting Information File 1 for full experimental data including general methods and chemicals, syntheses and characterization of complexes, procedures of catalytic tests and X-ray analysis.

## Supporting Information

File 1 General methods and chemicals, syntheses and characterization of complexes **8**–**10**, **13**, **14**, **16** and **17**, procedures of catalytic tests and X-ray analysis.
