# Supplementary material for: New aryloxybenzylidene ruthenium chelates – synthesis, reactivity and catalytic performance in ROMP
Source: Beilstein J Org Chem. 2015 Oct 14;11:1910–6. doi: 10.3762/bjoc.11.206 (PMC4660962; doi:10.3762/bjoc.11.206)

Supporting Information

for

New aryloxybenzylidene ruthenium chelates – synthesis, reactivity and catalytic performance in ROMP

Patrycja Żak, Szymon Rogalski, Mariusz Majchrzak, Maciej Kubicki and Cezary Pietraszuk*

Address: Adam Mickiewicz University in Poznań, Faculty of Chemistry, Umultowska 89b, 61-614 Poznań, Poland

Email - Cezary Pietraszuk - pietrasz@amu.edu.pl

*Corresponding author

General methods and chemicals, syntheses and characterization of complexes 8–10, 13, 14, 16 and 17, procedures of catalytic tests and X-ray analysis

1. General methods and chemicals

Unless mentioned otherwise, all operations were performed by using standard Schlenk techniques. ^{1}H and ^{13}C NMR spectra were recorded on a Varian 400 operating at 402.6 and 101.2 MHz, respectively. ^{31}P NMR spectra were recorded on a Mercury 300 operating at 121.5 MHz. GC analyses were carried out on a Varian CP-3800 (column: Rtx-5 30m I.D. 0.53 mm) equipped with TCD. Mass spectrometry analyses were performed using a Synapt G2-S mass spectrometer (Waters) equipped with an electrospray ion source and quadrupole-time-of-flight mass analyser. Isopropanol was used as a solvent. The measurement was performed in positive ion mode with a desolvation gas flow 600 L/h and a capillary voltage set to 4500 V with a flow rate of 100µL/min. Chemicals were obtained from the following sources: dichloromethane, benzene, toluene, methanol, tetrahydrofuran, ethyl acetate, pyridine, benzene-d_6, dichloromethane-d_2, second generation Grubbs' catalyst, decane, dodecane, triphenylphosphine, tri(*p*-tolyl)phosphine, tris(4-trifluoromethylphenyl)phosphine, tricyclohexylphosphine, 2-(prop-1-enyl)phenol, *n*-butyllithium (1.6 M), 2-hydroxy-5-*tert*-butylbenzaldehyde, 2-hydroxy-5-nitrobenzaldehyde, methyltriphenylphosphonium bromide, diethyl diallylmalonate, cyclooctadiene, allylbenzene, *Z*-1,4-bis(acetoxy)but-2-ene, HCl (2.0 M in Et_2O), calcium hydride and copper(I) chloride were purchased from Aldrich. Vinyl ethyl and *n*-hexane were provided from Chempur. 4-*tert*-butyl-2-vinylphenol, 4-nitro-2-vinylphenol were prepared according to the literature procedure [1]. The compound *exo*-*N*-2-ethylhexylnorbornene dicarboximide was synthesized according to the published procedure [2,3]. All solvents were dried over CaH_2 prior to use and stored under argon over 4 Å type molecular sieves. CH_2Cl_2 was additionally passed through

a column with alumina and after that it was degassed by repeated freeze-pump-thaw cycles.

2. Synthesis of aryloxybenzylidene–ruthenium complexes

2.1. Synthesis of 8. In a similar manner to a procedure in [1] a 50 mL flask equipped with a magnetic stirring bar and connected to an argon/vacuum line was charged under argon with 0.35 g (4.21×10^{-4} mol) of complex **4**, 0.1104 g (4.21×10^{-4} mol) of triphenylphosphine and 15 mL of dry dichloromethane. Then 60 μL (4.63×10^{-4} mol) of 2-(prop-1-enyl)phenol was added to the reacting mixture. The mixture was stirred at 40 °C for 24 h. After this time the solvent was evaporated under vacuum and *n*-hexane (5 mL) was added to the remaining contents to form a green precipitate. Then the resulted solution was decanted from the precipitate and the obtained solid was dried under vacuum (0.3074 g, isolated yield = 90%). *Spectroscopic data*: **^{1}H NMR** (C_6D_6, *δ*, ppm): 16.10 (s, 1H, Ru=C*H*), 7.38-7.29 (m, 6H, Ph), 7.00-6.98 (m, 10H, Ph, Ar), 6.83 (brs, 1H, Ar), 6.78 (brs, 1H, Ar), 6.75 (brs, 1H, Ar), 6.59 (d, J_{HH} = 8.5 Hz, 1H, Ar), 6.54 (dd, J_{HH} = 7.6, 1.2 Hz, 1H, Ar), 6.26-6.18 (m, 2H, Ar), 2.97-3.45 (overlapping multiplets, 4H, NCH_2CH_2N), 2.82 (s, 3H, mesityl CH_3), 2.79 (s, 3H, mesityl CH_3), 2.73 (s, 3H, mesityl CH_3), 2.12 (s, 3H, mesityl CH_3), 2.06 (s, 3H, mesityl CH_3), 1.58 (s, 3H, mesityl CH_3); **^{13}C NMR** (C_6D_6, *δ*, ppm): 17.07, 19.01, 19.16, 20.11, 21.08, 21.41, 51.09, 51.15, 111.17, 117.51, 122.43, 129.09, 129.22, 129.46, 129.87, 130.14, 130.43, 130.78, 130.91, 131.29, 134.37 (d, *J* = 10.7 Hz), 135.88, 136.64, 137.32, 137.46, 138.52, 138.84, 139.19, 148.21, 180.64, 221.49 (d, *J* = 75.0 Hz, Ru-*C*NN), 283.40 (Ru=*C*); **^{31}P NMR** (C_6D_6, *δ*, ppm): 43.27; **MS** (FD): m/z (%): 807.21 (25), 808.21 (36), 809.21 (55), 810.21 (100), 811.21 (51), 812.21 (70), 813.21 (30), 814.21 (18); **HRMS** (FD): calcd. for $C_{46}H_{46}ClN_2OPRu$: 810.2080; found: 810.2081;

2.2. Synthesis of complex 8 starting from dimer 16. A 25 mL flask equipped with a magnetic stirring bar and connected to an argon/vacuum line was charged under argon with 0.1 g (8.88×10^{-5} mol) of complex **16**, 0.0466 g (1.78×10^{-4} mol) of triphenylphosphine and 10 mL of dry benzene. The mixture was stirred at 80 °C for 1 h. After this time the solvent was evaporated under vacuum and *n*-hexane (5 mL) was added to the remaining contents to form a green precipitate. Then the resulted solution was decanted from precipitate and the obtained solid was dried under vacuum (0.1350 g, isolated yield = 94%).

2.3. Synthesis of 9. A 50 mL flask equipped with a magnetic stirring bar and connected to argon/vacuum line was charged under argon with 0.35 g (3.38×10^{-4} mol) of complex **5**, 0.1509 g (3.38×10^{-4} mol) of tris(4-trifluoromethylphenyl)phosphine and 15 mL of dry benzene. Then 48 μL (3.72×10^{-4} mol) of 2-(prop-1-enyl)phenol was added to the reacting mixture. The mixture was stirred at 60 °C for 3 h. After this time the solvent was evaporated under vacuum and methanol (5 mL) was added to the remaining contents to form a green precipitate. Then the resulted solution was decanted from precipitate and the obtained solid was dried under vacuum (0.2952 g, isolated yield = 86%).*Spectroscopic data*: **^{1}H NMR** (C_6D_6, *δ*, ppm): 15.79 (s, 1H, Ru=C*H*), 7.24 (s, 1H, Ar), 7.17 (d, J_{HH} = 2.9 Hz, 6H, C_6H_4-CF_3), 7.12 (d, J_{HH} = 2.9 Hz, 6H, C_6H_4-CF_3), 6.86 (s, 1H, Ar), 6.75 (s, 1H, Ar), 6.74 (s, 1H, Ar), 6.43 (d, J_{HH} = 7.7 Hz, 1H, Ar), 6.28 (d, J_{HH} = 8.6 Hz, 1H, Ar), 6.22 (s, 1H, Ar), 6.16 (t, J_{HH} = 7.1 Hz, 1H, Ar), 3.42-2.92 (m, 4H, NCH_2CH_2N), 2.73 (s, 3H, mesityl CH_3), 2.70 (s, 3H, mesityl CH_3), 2.68 (s, 3H, mesityl CH_3), 2.13 (s, 3H, mesityl

CH_3), 2.10 (s, 3H, mesityl CH_3), 1.43 (s, 3H, mesityl CH_3), **^{13}C NMR** (C_6D_6, δ, ppm): 16.84, 18.81, 19.08, 19.96, 21.05, 51.12 (br s), 111.84, 117.41, 122.31, 122.63, 122.76, 124.60 – 124.97 (m, CF_3), 128.89, 129.31, 129.57, 129.77, 130.33, 131.80, 132.64, 132.78, 134.45 (d, J = 11.5 Hz), 135.93, 136.35, 136.71, 137.24, 137.85, 139.00, 139.03, 139.39, 148.25, 180.75, 219.46 (d, J = 78.1 Hz, Ru-*C*NN), 282.41 (Ru=*C*); **^{31}P NMR:** (C_6D_6, δ, ppm): 44.97; **MS** (FD): m/z (%): 1011.17 (31), 1012.17 (38), 1013.17 (60), 1014.17 (100), 1015.17 (60), 1016.17 (74), 1017.17 (33), 1018.17 (20); **HRMS (FD)**: calc. for $C_{49}H_{43}ClF_9N_2OPRu$: 1014.1701; found: 1014.1686.

2.4. Synthesis of 10. A 50 mL flask equipped with a magnetic stirring bar and connected to an argon/vacuum line was charged under argon with 0.35 g (4.01×10^{-4} mol) of complex **6**, 0.1221 g (4.01×10^{-4} mol) of tri(*p*-tolyl)phosphine and 15 mL of dry benzene. Then 57 μL (4.42×10^{-4} mol) of 2-(prop-1-enyl)phenol was added to the reacting mixture. The mixture was stirred at 60 °C for 3 h. After this time the solvent was evaporated under vacuum and methanol (5 mL) was added to the remaining contents to form a green precipitate. Then the resulted solution was decanted from precipitate and the obtained solid was dried under vacuum (0.3014 g, isolated yield = 88%).Spectroscopic data: **^{1}H NMR** (C_6D_6, δ, ppm): 16.17 (s, 1H, Ru=C*H*), 7.31 (dd, J_{HH} = 10.1, 8.1 Hz, 6H, C_6H_4-CH_3), 6.97 (br s, 1H, Ar), 6.95 – 6.90 9m, 2H, Ar), 6.88 (s, 1H, Ar), 6.79 (pseudo d, 6H, C_6H_4-CH_3), 6.69 (d, J_{HH} = 8.3 Hz, 1H, Ar), 6.58 (dd, J_{HH} = 7.7, 1.2 Hz, 1H, Ar), 6.20 – 6.25 (m, 2H, Ar), 3.49-3.00 (m, 4H, NCH_2CH_2N), 2.88 (s, 3H, mesityl CH_3), 2.81 (s, 3H, mesityl CH_3), 2.72 (s, 3H, mesityl CH_3), 2.12 (s, 3H, mesityl CH_3), 2.10 (s, 3H, mesityl CH_3), 1.98 (s, 9H, C_6H_4-CH_3), 1.62 (s, 3H, mesityl CH_3); **^{13}C NMR** (C_6D_6, δ, ppm): 17.10, 19.07, 19.20, 20.17, 21.30, 21.23 (C_6H_4-*C*H_3), 21.37, 51.02 – 51.25, 111.05, 117.56, 122.40, 128.55, 128.65, 129.21, 129.45, 129.89, 130.38, 130.44, 134.26, 134.38 (d, J = 11.2 Hz),

136.68, 136.72, 137.36, 137.41, 138.54, 138.80, 138.82, 138.88, 139.29, 180.57, 222.04 (d, J = 74.8 Hz, Ru-*C*NN), 283.70 (Ru=*C*); **^{31}P NMR:** (C_6D_6, δ, ppm): 40.88; **MS** (FD): m/z (%): 849.25 (22), 850.25 (32), 851.25 (52), 852.25 (100), 853.25 (52), 854.25 (68), 855.26 (31), 856.25 (14); **HRMS** (FD): calc. for $C_{49}H_{52}ClN_2OPRu$: 852.2548; found: 852.2549;

2.5. Synthesis of 13. A 50 mL flask equipped with a magnetic stirring bar and connected to argon/vacuum line was charged under argon with 0.35 g (4.21×10^{-4} mol) of complex **4**, 0.1104 g (4.21×10^{-4} mol) of triphenylphosphine and 15 mL of dry benzene. Then 0.088 g (4.63×10^{-4} mol) of 4-*tert*-butyl-2-vinylphenol was added to the reacting mixture. The mixture was stirred at 60 °C for 3 h. After this time the solvent was evaporated under vacuum and methanol (5 mL) was added to the remaining contents to form a green precipitate. Then the resulted solution was decanted from precipitate and the obtained solid was dried under vacuum (0.3285 g, isolated yield = 90%). *Spectroscopic data:* **^{1}H NMR** (C_6D_6, δ, ppm): 15.93 (s, 1H, Ru=C*H*), 7.31 – 7.38 (m, 6H, Ph), 6.98 (dd, J_{HH} = 8.1, 2.1 Hz, 1H, Ar), 6.86 – 6.95 (m, 9H, Ph), 6.85 (br s, 1H, Ar), 6.81 (br s, 1H, Ar), 6.79 (br s, 1H, Ar), 6.49 (d, J_{HH} = 6.9 Hz, 1H, Ar), 6.47 (s, 1H, Ar), 6.38 (s, 1H, Ar), 3.45-3.01 (m, 4H, NCH_2CH_2N), 2.81 (s, 3H, mesityl CH_3), 2.79 (s, 6H, mesityl CH_3), 2.20 (s, 3H, mesityl CH_3), 2.07 (s, 3H, CH_3), 1.45 (s, 3H, mesityl CH_3), 1.28 (s, 9H, $C(CH_3)_3$); **^{13}C NMR** (C_6D_6, δ, ppm): 16.88, 18.98, 19.45, 20.09, 21.38, 31.86 ($C(CH_3)_3$), 33.45 ($C(CH_3)_3$), 50.94, 51.24, 117.10, 117.99, 128.99, 129.11, 129.17, 129.30, 129.44, 129.85, 130.36, 130.87, 131.24, 133.32, 133.95, 134.40 (d, J = 10.8 Hz), 136.89, 136.97, 137.16, 137.71, 138.48, 139.18, 147.89, 179.56, 221.57 (d, J = 76.1 Hz, Ru-*C*NN), 282.04 (Ru=*C*); **^{31}P NMR** (C_6D_6, δ, ppm): 43.41; **MS** (FD): m/z (%): 863.27 (25), 864.27 (37),

865.27 (56), 866.27 (100), 867.27 (54), 868.27 (74), 869.27 (31), 870.27 (18); **HRMS** (FD): calcd. for $C_{50}H_{54}ClN_2OPRu$: 866.2693; found: 866.2706;

2.6. Synthesis of 14. A 50 mL flask equipped with a magnetic stirring bar and connected to an argon/vacuum line was charged under argon with 0.35 g (4.21×10^{-4} mol) of complex **4**, 0.1104 g (4.21×10^{-4} mol) of triphenylphosphine and 15 mL of dry benzene. Then 0.076 g (4.63×10^{-4} mol) of 4-nitro-2-vinylphenol was added to the reacting mixture. The mixture was stirred at 60 °C for 3 h. After this time the solvent was evaporated under vacuum and *n*-hexane (5 mL) was added to the remaining contents to form an orange precipitate. Then the resulted solution was decanted from precipitate and the obtained solid was dried under vacuum (0.3315 g, isolated yield = 92 %). *Spectroscopic data*: ^{1}**H NMR** (C_6D_6, δ, ppm): 16.56 (s, 1H, Ru=C*H*), 7.96 (dd, J_{HH} = 9.4, 2.5 Hz, 1H, Ar), 7.69 (d, J_{HH} = 2.5 Hz, 1H, Ar), 7.31 - 7.21 (m, 6H, Ar), 7.1 (s, 1H, Ar), 6.93 – 6.81 (m, 9H, Ph), 6.66 (s, 1H, Ar), 6.63 (s, 1H, Ar), 6.20 (d, J_{HH} = 9.2 Hz, 1H, Ar), 5.93 (s, 1H, Ar), 3.39-2.88 (m, 4H, NCH_2CH_2N), 2.70 (s, 3H, mesityl CH_3), 2.67 (s, 3H, mesityl CH_3), 2.58 (s, 3H, mesityl CH_3), 2.15 (s, 3H, mesityl CH_3), 2.02 (s, 3H, mesityl CH_3), 1.46 (s, 3H, mesityl CH_3); ^{13}**C NMR** (C_6D_6, δ, ppm):17.01, 18.71, 18.72, 20.04, 20.89, 21.40, 50.86-50.85, 51.37-51.53, 115.91, 117.86, 125.70, 129.31, 129.42, 129.56, 129.58, 129.61, 129.96, 130.01, 130.59, 133.78, 134.07 (d, *J* = 11.0 Hz), 134.46, 135.77, 136.13, 137.00, 137.97, 138.89, 139.17, 139.33, 145.54, 183.78, 219.78 (d, *J* = 72.8 Hz, Ru-*C*NN), 286.62 (Ru=*C*); ^{31}**P NMR** (C_6D_6, δ, ppm): 42.17; **MS** (FD): m/z (%): 852.20 (26), 853.19 (40), 854.20 (60), 855.19 (100), 856.20 (68), 857.19 (73), 858.20 (44), 859.20 (21); **HRMS** (FD): calc. for $C_{46}H_{45}ClN_3O_3PRu$: 855.1917; found: 855.1931

2.7. Synthesis of 16. A 25 mL flask equipped with a magnetic stirring bar and connected to an argon/vacuum line was charged under argon with 0.15 g (1.85×10^{-4} mol) of complex **8**, 0.0896 g (1.85×10^{-4} mol) of copper(I) chloride and 10 mL of dry benzene. The mixture was stirred at 60 °C for 24 h. After this time the solvent was decanted from precipitate and placed in a second flask. The solvent was evaporated under vacuum and *n*-hexane (5 mL) was added to the remaining contents to form a green precipitate. Then the resulted solution was decanted from precipitate and the obtained solid was washed three times with n-hexane (3 × 5 mL) and dried under vacuum (0.3006 g, isolated yield = 88%).

2.8. Synthesis of 17. A 25 mL flask equipped with a magnetic stirring bar and connected to an argon/vacuum line was charged under argon with 0.06 g (7.4×10^{-5} mol) of **8** and 5 mL of dry dichloromethane. Then the 37 μL (7.4×10^{-5} mol) of 2.0 M solution of hydrogen chloride in diethyl ether was added to the reacting mixture. The reaction mixture was stirred at room temperature for 5 min, during which time the solution changed colour from dark-green to a light-green. After this time the solvent and residual acid were evaporated under vacuum and 5 mL of dry *n*-hexane was added to the remaining content to form a green precipitate. Next the resulted solution was decanted from precipitate and obtained green microcrystalline solid was washed in hexane and dried under vacuum (isolated yield 0.059 g, 95%). *Spectroscopic data*: **^{1}H NMR** (C_6D_6, *δ*, ppm): 16.51 (s, 1H, Ru=C*H*), 7.37-7.29 (m, 6H, Ph), 6.96-6.85 (m, 10H, Ph, Ar), 6.82 (brs, 1H, Ar), 6.70 (brs, 2H, Ar), 6.52 (d, J_{HH} = 7.6 Hz, 1H, Ar), 6.23 (t, J_{HH} = 7.1 Hz, 1H, Ar), 6.08 (brs, 1H, Ar), 3.48-2.99 (overlapping multiplets, 4H, NCH_2CH_2N), 2.93 (s, 3H, mesityl CH_3), 2.69 (s, 3H, mesityl CH_3), 2.63 (s, 3H,

mesityl CH_3), 2.09 (s, 3H, mesityl CH_3), 2.04 (s, 3H, mesityl CH_3), 1.73 (s, 3H, mesityl CH_3); **^{31}P NMR** (C_6D_6, δ, ppm): 43.73.

3. Procedures of catalytic tests

3.1. ROMP of COD. In a similar manner to a procedure in [1] the oven-dried 5 mL glass reactor equipped with a condenser and magnetic stirring bar was charged under argon with 2 mL of CH_2Cl_2, 100 µL of cyclooctadiene (8.04×10^{-4} mol) and 80 µL of dodecane (internal standard). The reaction mixture was placed in an oil bath and preheated at 40 °C. Then the catalyst complex (7.24×10^{-7} mol) was added under argon. The mixture was heated at 40 °C under argon. After the given reaction time 30 µL of the reaction mixture was taken, placed in a 1 mL vial and quenched by the addition of 15 µL vinyl ethyl ether and analysed by gas chromatography. The conversion of the substrates was calculated using the internal standard method.

3.2. ROMP of COD with HCl as an activating agent. The oven dried 5 mL glass reactor equipped with a condenser and magnetic stirring bar was charged under argon with 2 mL of CH_2Cl_2, 100 µL of cyclooctadiene (8.04×10^{-4} mol) and 80 µL of dodecane (internal standard). The reaction mixture was placed in an oil bath and preheated at 40 °C. Then (7.24×10^{-7} mol) of catalyst and HCl (2 M in Et_2O) (1.49×10^{-6} mol) were added consecutively under argon. The mixture was heated at 40 °C under argon. After the given reaction time 30 µL of the reaction mixture was taken, placed in a 1 mL vial and quenched by the addition of 15 µL vinyl ethyl ether and analyzed by gas chromatography. The conversion of the substrates was calculated using the internal standard method.

3.3. ROMP of monomer 15. The reactions were carried out under inert conditions, using a Wilmad LPV NMR tube. In a typical protocol the ruthenium initiator (1.915×10^{-7} mol) and monomer **15** (10 mg, 3.83×10^{-5} mol) were weighed into sample vials and dissolved in $CDCl_3$ (0.5 mL, dry). The solution of monomer **15** was transferred into the NMR tube. Then, the solution of ruthenium initiator was added, mixed (molar ratio: [**15**]:[cat.] = 200:1) and activated with HCl (2.0 M in Et_2O) (3.83×10^{-7} mol). The reactions were carried out at room temperature and at 40 °C. The course of the reactions was monitored by gas chromatography and ^{1}H NMR spectroscopy.

4. X-ray analysis. X-ray diffraction data were collected at 130(1) K by the ω-scan technique, on an Agilent Technologies four-circle SuperNova diffractometer equipped with an Atlas detector [4] and equipped with a Nova microfocus CuK_α radiation source (λ = 1.54178 Å). The temperature was controlled with an Oxford Instruments Cryosystem device. The data were corrected for Lorentz-polarization effects as well as for absorption (multiscan) [4]. Accurate unit-cell parameters were determined by a least-squares fit of and 11114 reflections of highest intensity, chosen from the whole experiment. The calculations were mainly performed within the WinGX program system [5]. The structures were solved with SIR92 [6] and refined with the full-matrix least-squares procedure on F^2 by SHELXL97 [7]. Scattering factors incorporated in SHELXL97 were used. The function $\Sigma w(|F_o|^2 - |F_c|^2)^2$ was minimized, with $w^{-1} = [\sigma^2(F_o)^2 + (0.058 \cdot P)^2 + 1.8055 \cdot P]$, where $P = [\mathrm{Max}(F_o^2, 0) + 2F_c^2]/3$. All non-hydrogen atoms were refined anisotropically, hydrogen atoms from methyl groups were placed geometrically, in idealized positions, and refined as riding group with their U_{iso}'s set at 1.5 times U_{eq} of appropriate carrier atom; all other hydrogen atoms

were found in difference Fourier maps and isotropically refined. Crystallographic data (excluding structure factors) for the structural analysis has been deposited with the Cambridge Crystallographic Data Centre, No. CCDC 891681. Copies of this information may be obtained free of charge from: The Director, CCDC, 12 Union Road, Cambridge, CB2 1EZ, UK. Fax: +44(1223)336-033, e-mail:deposit@ccdc.cam.ac.uk, or www: www.ccdc.cam.ac.uk. Crystal data: $C_{56}H_{62}Cl_2N_4O_2Ru_2$, M_r = 1096.14, monoclinic, $P2_1/c$, a = 11.5309(2) Å, b = 16.1759(3) Å, c = 13.5476(2) Å, β = 100.962(1)°, V = 2480.83(7) $Å^3$, Z = 2, F(000) = 1128, d_x = 1.47 g cm^{-3}, μ = 6.28 mm^{-1}. 14928 reflections collected up to 2Θ = 147.8°, of which 4916 independent (R_{int}=0.031), 4802 with I>2σ(I). Final R(I>2σ(I)) = 0.032, wR2(I>2σ(I))-0.087, R (all data) = 0.033, wR2(all data) = 0.087, S = 1.02, max/min Δρ = 0.61/−1.38 e $Å^{-3}$.

References

1. Żak, P.; Rogalski, S.; Przybylski, P.; Kubicki, M.; Pietraszuk, C. *Eur. J. Inorg. Chem.* **2014,** 1131–1136.
2. Khosravi, E.; Al-Hajaji, A. A. *Eur. Polym. J.* **1998,** *34,* 153-157.
3. Hine, P. J.; Leejarkpai, T.; Khosravi, E.; Duckett, R. A.; Feast, W. J. *Polymer* **2001,** *42*, 9413–9422.
4. CrysAlis PRO, Agilent Technologies, 2010.
5. Farrugia, L. J. *J. Appl. Cryst.* **1999,** *32*, 837–838.
6. Altomare, A.; Cascarano, G.; Giacovazzo, C.; Guagliardi, A. *J. Appl. Cryst.* **1993,** 26, 343–350.
7. Sheldrick, G. M. *Acta Cryst.* **2015,** C71, 3–8.

4. ^{1}H and ^{31}P NMR spectra of complexes

4.1. Complex 8

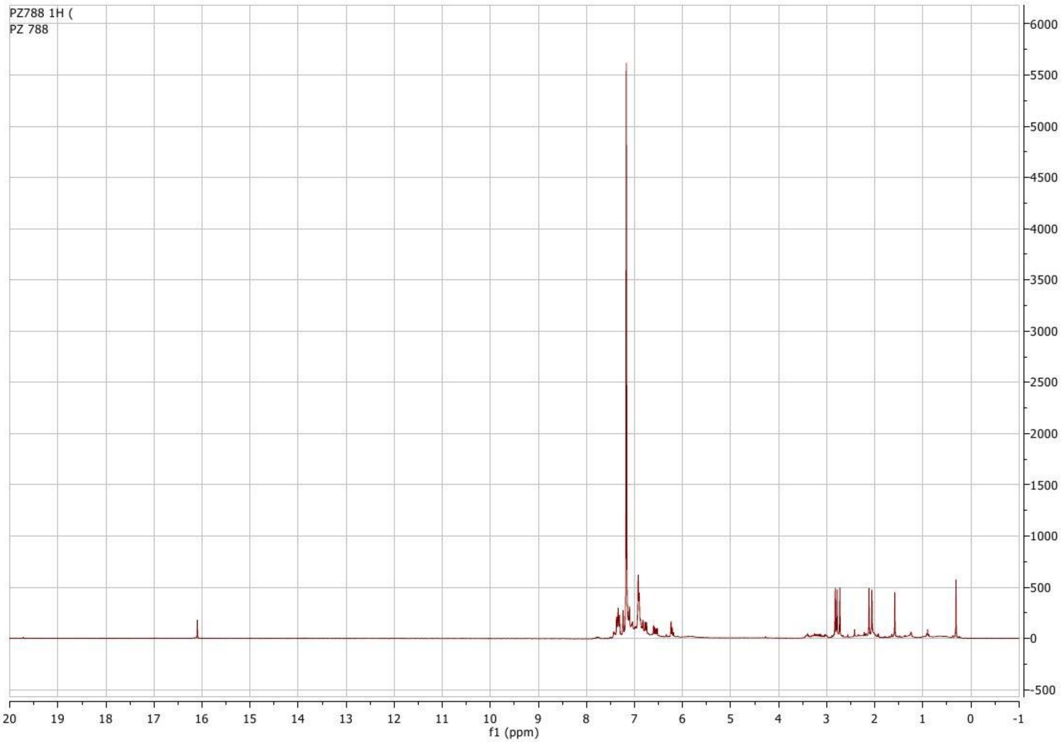

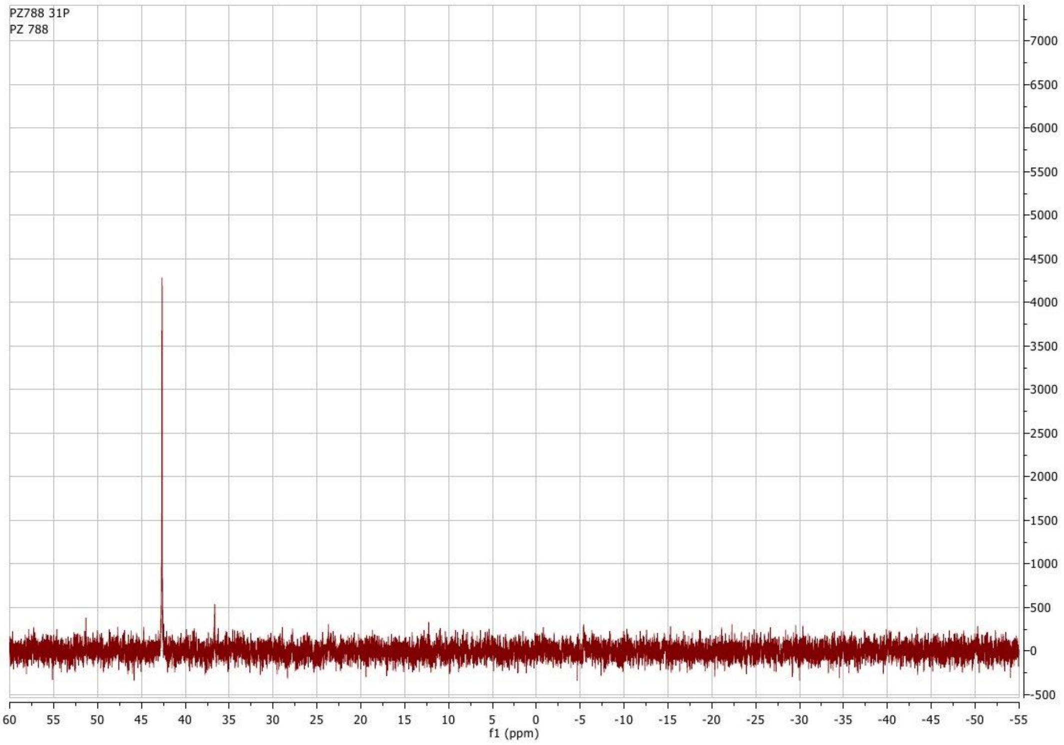

4.2. Complex 9

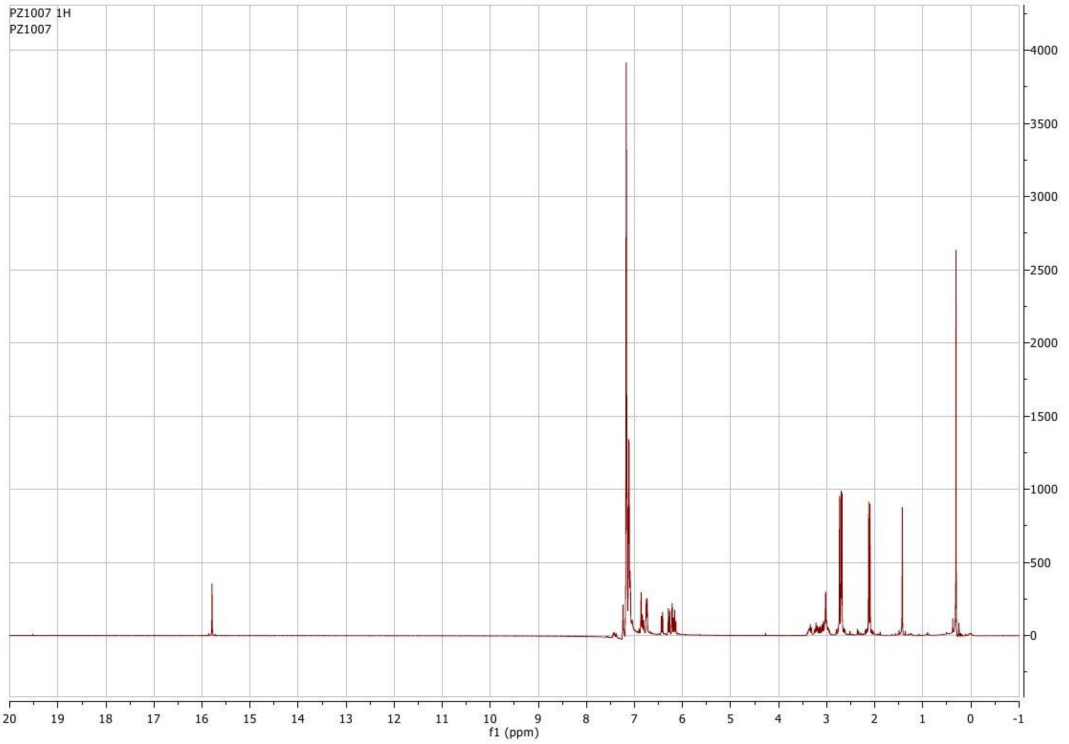

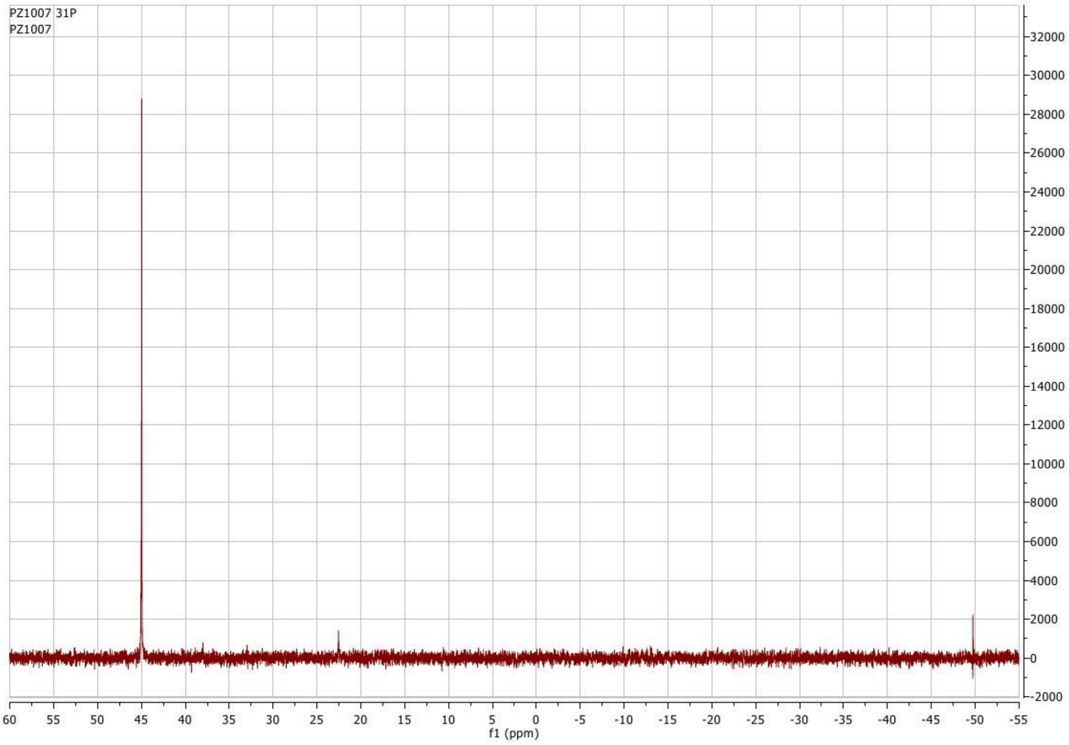

4.3. Complex 10

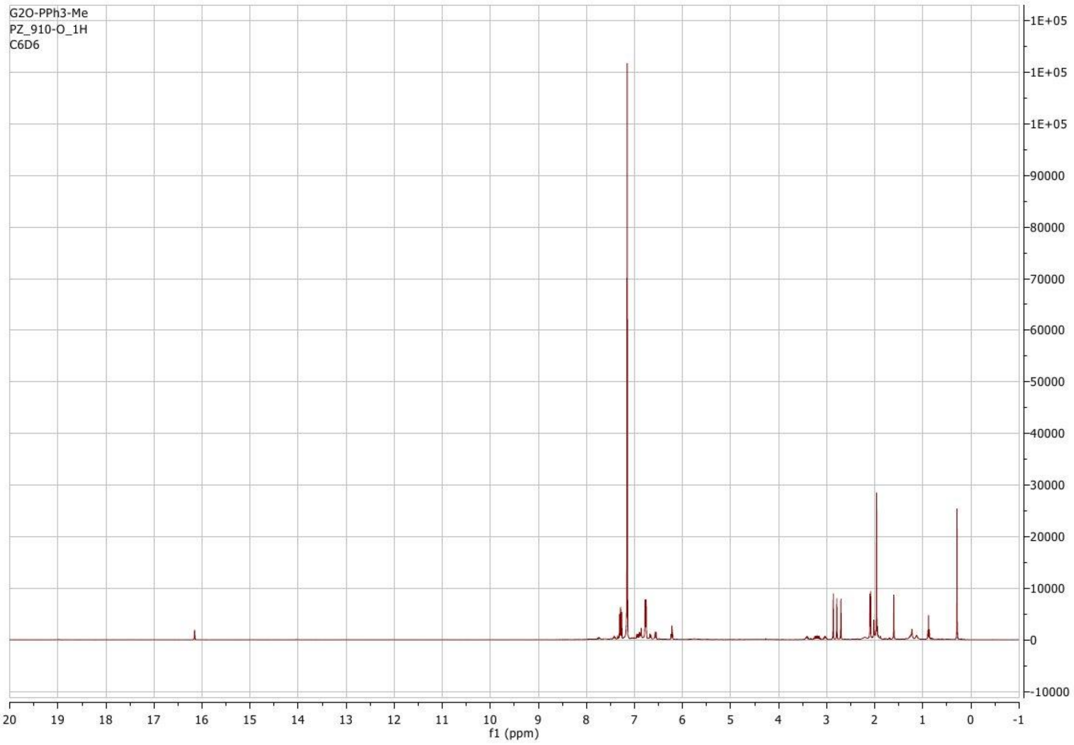

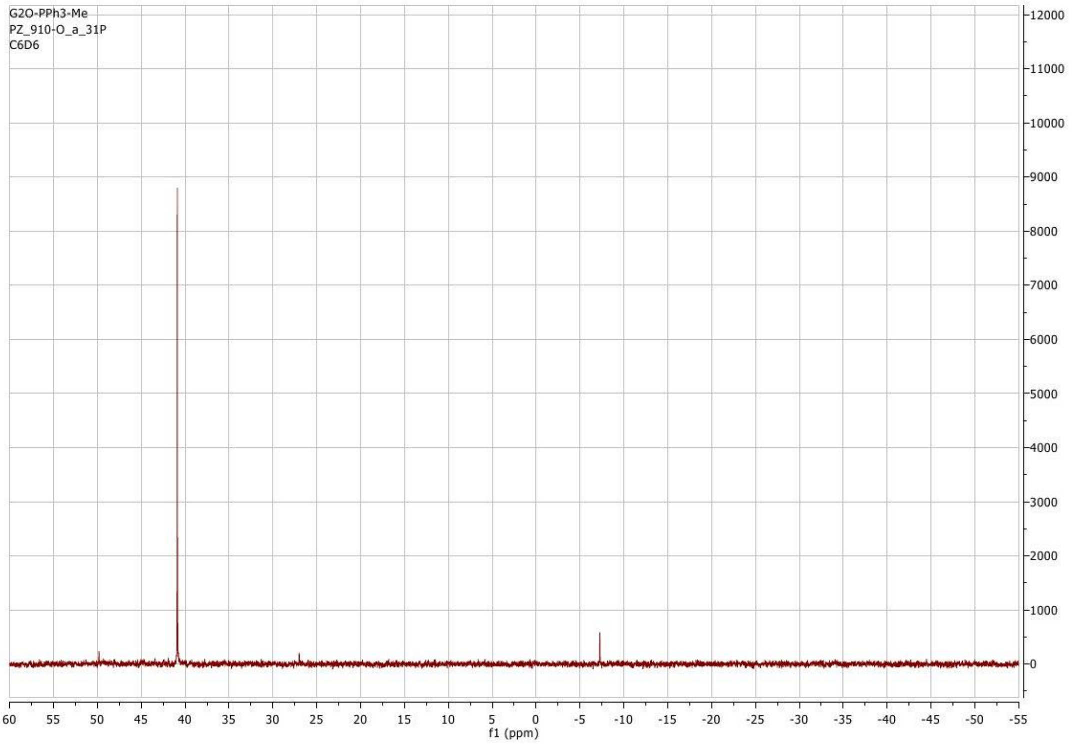

4.4. Complex 13

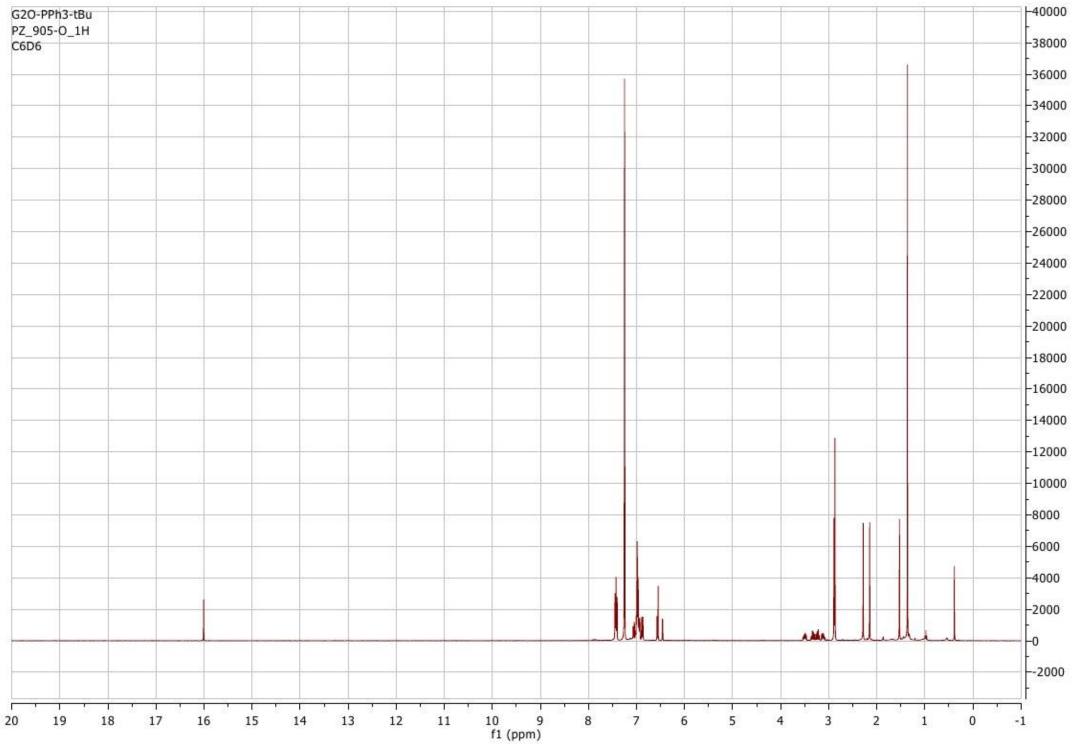

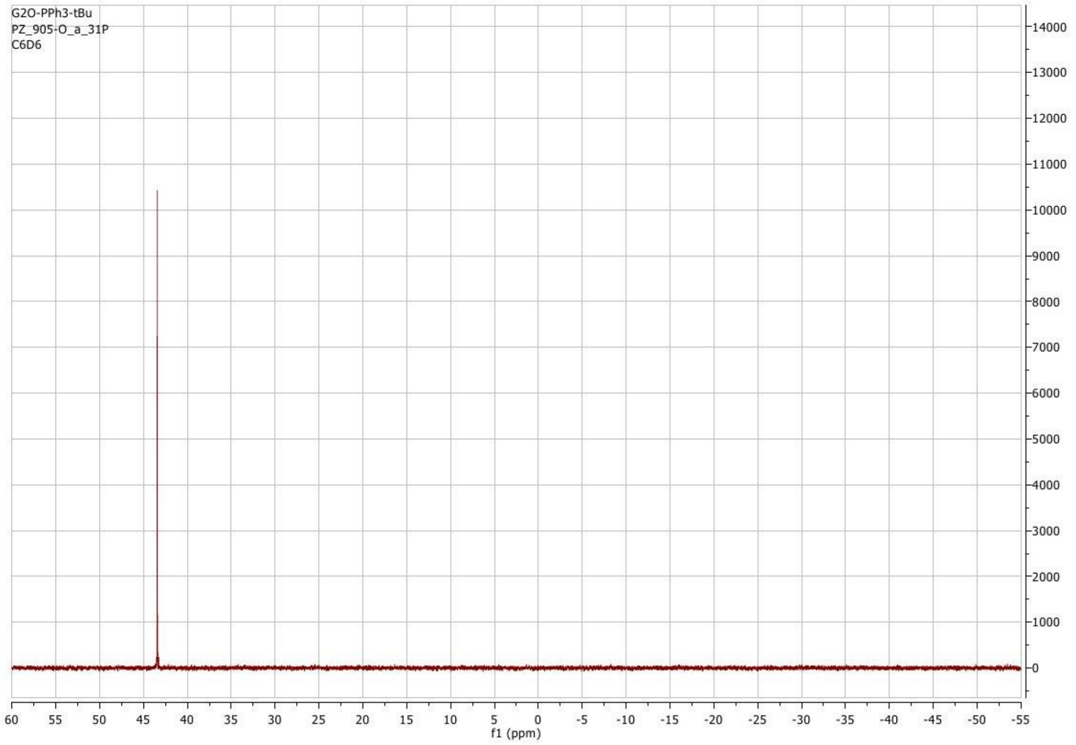

4.5. Complex 14

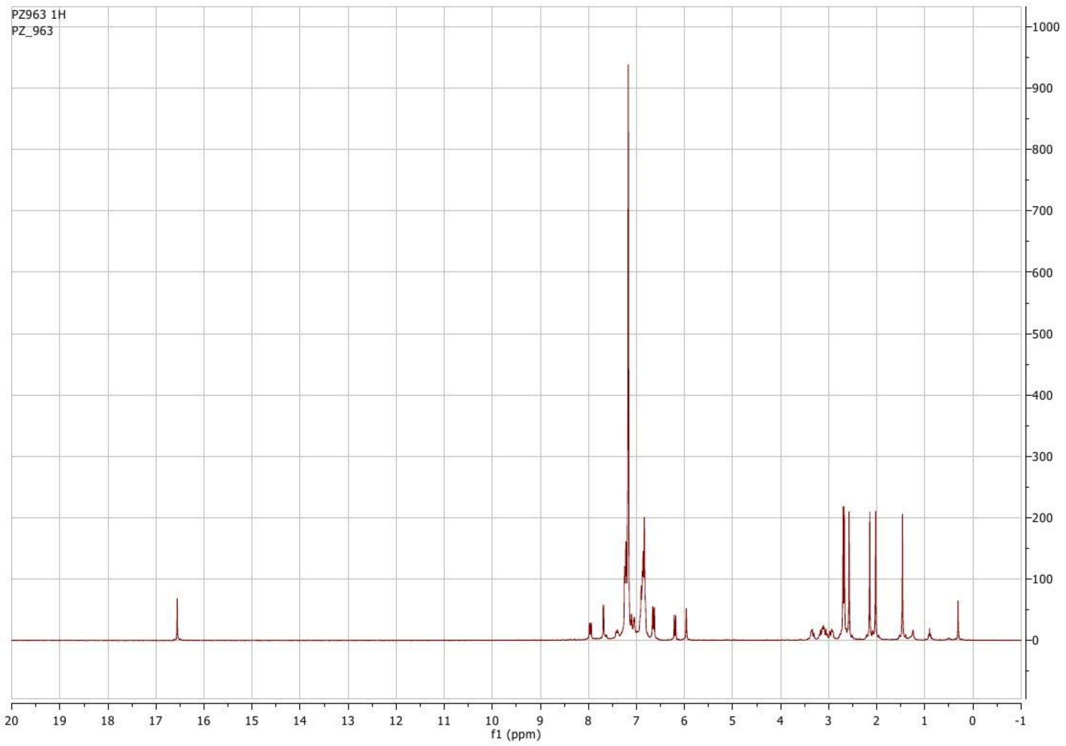

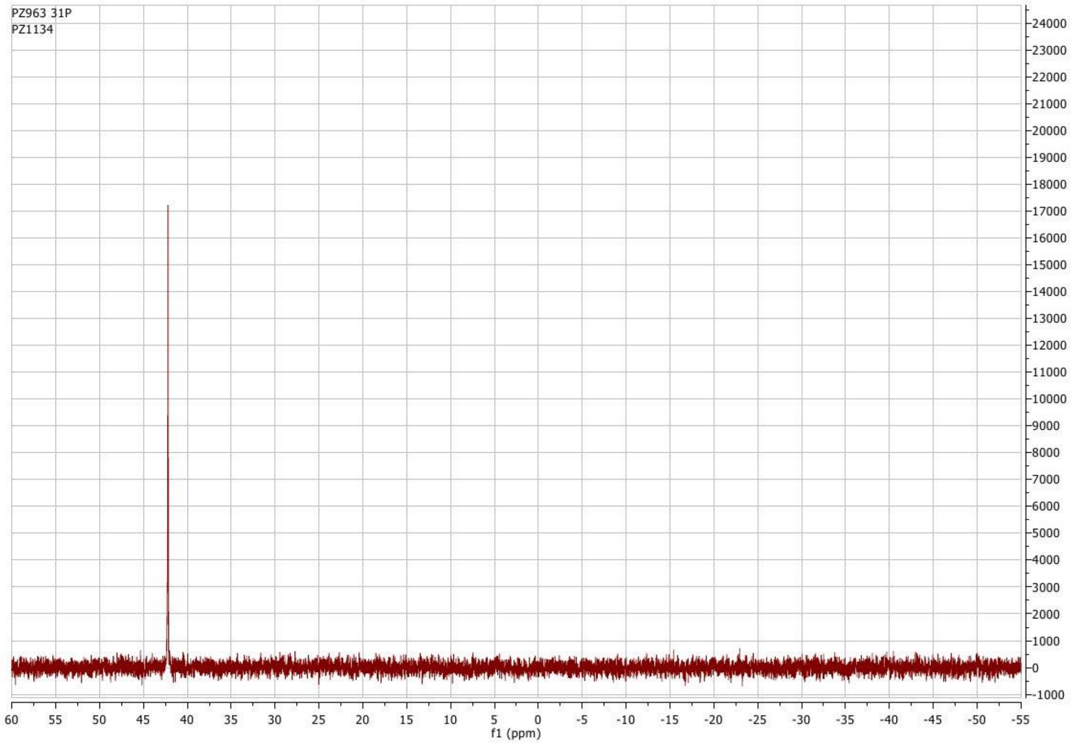

4.6. Complex 17

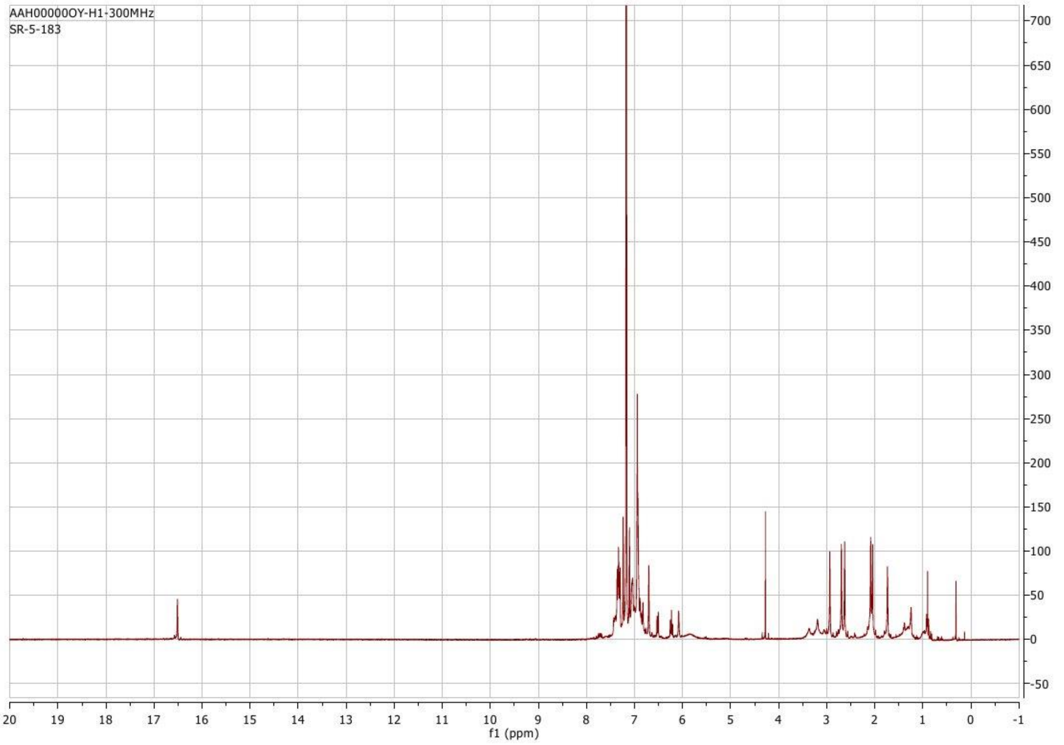

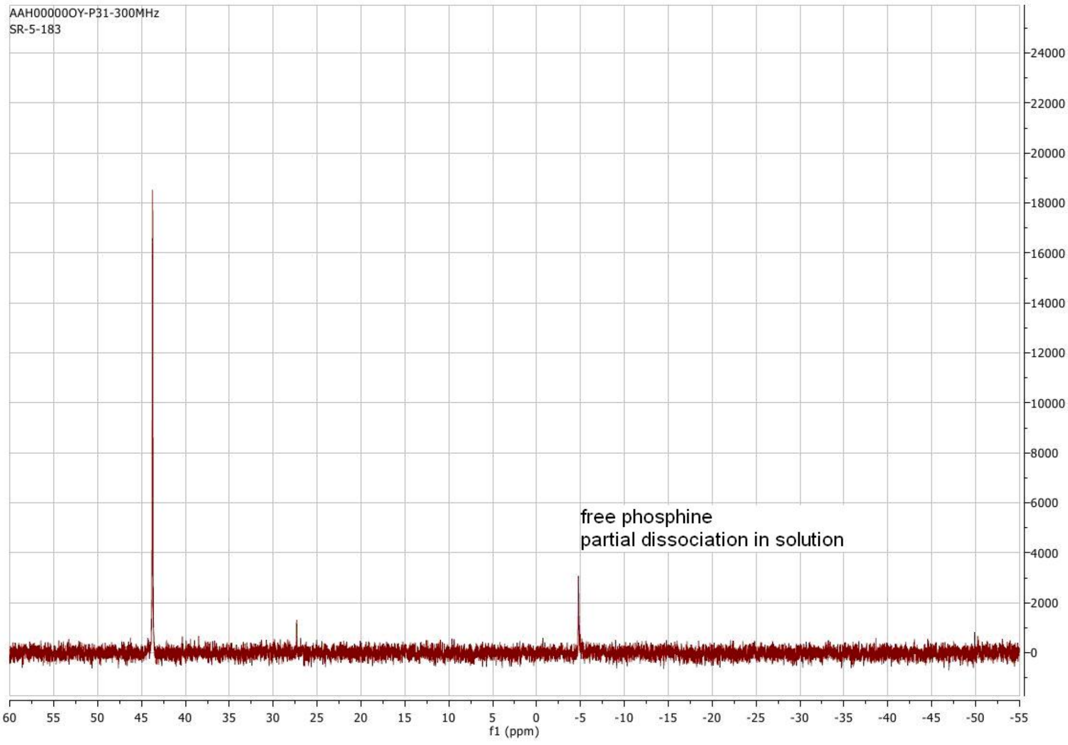

Supplement: File 1 — General methods and chemicals, syntheses and characterization of complexes 8–10, 13, 14, 16 and 17, procedures of catalytic tests and X-ray analysis. [file Beilstein_J_Org_Chem-11-1910-s001.pdf]
